# Time to Recovery From Severe Acute Malnutrition and Its Predictors Among Children Aged 6–59 Months in Ethiopia: A Bayesian Joint Modeling Approach

**DOI:** 10.1111/mcn.70220

**Published:** 2026-07-28

**Authors:** Dejen Kahsay Asgedom, Maru Feleke, Abraham Walelegn Zelalem, Solomon Keflie Assefa, Bewuketu Terefe, Nebiyu Mekonnen Derseh, Ayenew Molla Lakew

**Affiliations:** ^1^ Department of Public Health, College of Medicine and Health Sciences Samara University Samara Ethiopia; ^2^ Department of Epidemiology and Biostatistics, Institute of Public Health, College of Medicine and Health Sciences University of Gondar Gondar Ethiopia; ^3^ Department of Psychiatry, College of Medicine and Health Science Dire‐Dawa University Dire‐Dawa Ethiopia; ^4^ Department of Public Health Pawi Health Science College Pawi Ethiopia; ^5^ College of Medicine and Health Science University of Gondar Gondar Ethiopia; ^6^ School of Public Health The University of Queensland Brisbane Australia

**Keywords:** Bayesian joint model, children aged 6–59 months, Ethiopia, severe acute malnutrition, time to recovery (TTR), weight changes

## Abstract

In low‐resource settings such as Ethiopia, where predictors of time to recovery from SAM remain understudied, identifying predictors and longitudinal weight patterns associated with time to recovery (TTR) from SAM is crucial for early and appropriate clinical interventions. Therefore, this study aimed to determine the incidence and predictors of recovery from SAM, as well as its association with longitudinal weight changes, among children aged 6–59 months in Ethiopia. An institution‐based retrospective follow‐up study was conducted among SAM children aged 6–59 months at Amhara Region Comprehensive Specialized Referral Hospitals (CSEHs), Northwest Ethiopia. Cox PH and linear mixed effect models were jointly used to evaluate the effects of time‐dependent weight trajectories on the time to recovery from the SAM via the Bayesian approach. An adjusted hazard ratio (AHR) with a 95% credible interval (CrI) was used to determine statistically significant predictors. The data were entered into the Kobo toolbox and analyzed with R software version 4.5.1. A total of 480 children aged 6–59 months were followed for 4356 person–days of observation (PDO). At the end of the follow‐up, 291 patients (291/480, 60.6%) had recovered, with an incidence density of 6.69 cases per 100 PDOs (95% CI: 5.93–7.47). The median recovery time from severe acute malnutrition was 10 days (IQR: 7–16). The time‐dependent current value of weight was significantly positively associated with the time to recovery (AHR = 1.15 95% CrI: [1.04, 1.26]). Additionally, being HIV seropositive (AHR = 0.46, 95% CrI: [0.22, 0.90]), having edematous malnutrition (AHR = 0.48, 95% CrI: [0.32, 0.70]), vomiting at admission (AHR = 0.62, 95% CrI: [0.44, 0.87]), anemia at admission (AHR = 0.45, 95% CrI: [0.31, 0.64]), folic acid supplementation (AHR = 0.70, 95% CrI: [0.53, 0.94]), F‐100 formula milk (AHR = 1.40, 95% CrI: [1.08, 1.80]), and antibiotic use (AHR = 1.58, 95% CrI: [1.04, 2.46]) were significant predictors of the time to recovery from severe acute malnutrition. The proportion of recovery reported in this study was lower than the minimum 75% threshold set by the national and international SPHERE standards. The median recovery time of 10 days was optimal. The unobserved true current value of weight change was significantly associated with the time to recovery from severe acute malnutrition. Additionally, HIV status, anemia, edema, vomiting at admission, folic acid supplementation, F‐100 formula milk, and IV antibiotic use were significant predictors of the time to recovery from SAM. F‐100 supplements and IV antibiotics should be used to improve and increase the recovery rate.

## Summary

- The proportion of recovery reported in this study was lower than the SPHERE standards.
- The unobserved true current value of weight change was significantly associated with the time to recovery from severe acute malnutrition. Therefore, health professional should strengthen routine weight monitoring as a key biomarker to identify SAM complications early and reduce their long‐term effects.
- F‐100 supplements and IV antibiotics should be used to improve and increase the recovery rate.
- Children with HIV/AIDS, anemia, edema, vomiting at admission, and folic acid supplementation should be closely monitored and receive additional medical care to improve the recovery rate.

**Abbreviations**
AHR: adjusted hazard ratio
CSRHs: comprehensive specialized referral hospital
HIV/AIDS: human immunodeficiency virus/acquired immune deficiency syndrome
IQR: interquartile range
IV: intravenous
MCMC: Markov chain Monte Carlo
MUAC: mid‐upper arm circumference
SAM: severe acute malnutrition
TB: tuberculosis
WHO: World Health Organization

## 1 Introduction

According to the World Health Organization (WHO), severe acute malnutrition (SAM) in children aged 6–59 months is characterized by an extremely low weight‐for‐height or weight‐for‐length ratio, the presence of bilateral pitting edema, or a significantly reduced mid‐upper arm circumference (WHO 2023). SAM is an important public issue in low‐ and middle‐income countries, contributing to high rates of illness, death, and long‐term disability (2023). Globally, approximately 19 million children under 5 years of age were affected by SAM in 2023 (Organization, W. H. 2023). In 2022, approximately 45 million children under 5 years of age (6.8%) suffered from wasting, including 13.6 million (2.1%) with severe wasting (WHO 2023). Over 75% of severely wasted children live in Asia, whereas an additional 22% live in Africa (WHO 2023).

SAM remains a critical public health challenge in Ethiopia. According to the 2023 Food and Nutrition Survey (FNS), 11% of children under 5 years of age suffer from acute malnutrition (wasting), whereas 39% exhibit stunted growth (WHO 2023). The prevalence of wasting ranges from 4% in Addis Ababa to 26% in the Afar region in relation to climate shocks, conflicts, and disease outbreaks (WHO 2023).

Poor management of undernutrition in early childhood can result in long‐term consequences, including impaired cognitive development, poorer educational performance, and reduced economic productivity in adulthood (Oddy et al. 2003; Victora et al. 2008). Globally, SAM contributes to approximately 400,000 child deaths annually (WHO 2023), whereas in Ethiopia, it ranks among the top three causes of underfive mortality, alongside pneumonia and neonatal sepsis, accounting for 20% of pediatric hospital admissions (Bizuneh et al. 2022).

In Ethiopia, the recovery rate among children aged 6–59 months from SAM ranges from 43.6% (Tirore et al. 2017) to 90.9% (Husen et al. 2022). The international treatment protocols agree that SAM patients admitted to stabilization centers (STCs) should achieve recovery within 1 month (Sphere Association 2018). However, Ethiopian studies report median recovery durations exceeding this benchmark, with reported periods spanning from 9 days (Workie et al. 2025) to 8.7 weeks (Atnafe et al. 2019). Additionally, the global standard for SAM treatment success requires at least a 75% recovery rate. However, available data indicate that Ethiopia's performance against this target has been inconsistent, with documented recovery rates of 43.6%, 87%, and 90.9% across different studies (Teferi, Lera et al. 2010; Desyibelew et al. 2017; Husen et al. 2022).

The predictors for time to recovery (TTR) from SAM among children aged 6–59 months were age of the child (Tadesse et al. 2021), residence (Wondmeneh and Giruma 2025), the presence of pneumonia and/or tuberculosis (Tadesse et al. 2021; Aye et al. 2023; Mekonnen et al. 2025), HIV/AIDS (Bizuneh et al. 2022; Mekonnen et al. 2025; Mekonnen et al. 2025), SAM type (Bizuneh et al. 2022; Workie et al. 2025), vaccination status (Mekonnen et al. 2025; Workie et al. 2025), deworming (Getahun et al. 2024; Mekonnen et al. 2025), anemia (Tadesse et al. 2021), the presence of diarrhea and/or vomiting (Tadesse et al. 2021; Husen et al. 2022; Getahun et al. 2024), breast feeding status (Getahun et al. 2024; Mekonnen et al. 2025), antibiotic use (Getahun et al. 2024; Elema et al. 2025), F‐100 milk supplements (Fikrie et al. 2019; Bizuneh et al. 2022), and nasogastric intubation (Oumer et al. 2021; Bizuneh et al. 2022), as documented in previous studies. Body weight is a crucial clinical biomarker for diagnosing and managing SAM in children under 5 years of age and plays a key role in monitoring disease progression and preventing complications (Choudhury et al. 2018; Gebremedhin et al. 2020). Studies have also revealed that weight changes are strongly correlated with TTR from SAM among children aged 6–59 months and that optimal daily weight monitoring can shorten the cure time and reduce the risk of SAM complications (Teferi, Lera et al. 2010; Asres et al. 2018; Atnafe et al. 2019). However, little is known about the associations between longitudinal weight trajectories and the TTR from SAM.

The 65th World Health Assembly set a target to reduce the prevalence of childhood wasting to less than 5% by 2025 and less than 3% by 2030 (WHO 2023). To evaluate this target, current and up‐to‐date data on recovery time are crucial. In addition, the findings of the current study are important for identifying potential determinants that will be valuable for program managers to produce appropriate clinical interventions.

Previous studies conducted in Ethiopia used separate analyses (Atnafe et al. 2019; Baraki et al. 2020; Bizuneh et al. 2022), which ignored the dependency and association between longitudinal weight change and TTR. A joint modeling approach is preferred over a separate survival model (Hickey et al. 2016), as it provides more efficient estimates of the treatment effects on the TTR and longitudinal markers of weight change measured with error. Hence, this study uses a joint modeling approach to account for the dependence and association of TTR and weight change.

The Bayesian modeling approach offers several advantages, such as incorporating expert knowledge through prior distributions for model parameters, enabling the fitting of more complex models. Unlike the classical approach, it does not rely on asymptotic results (e.g., the asymptotic normality of maximum likelihood estimates). Additionally, it provides more precise inference through simulation techniques such as Markov chain Monte Carlo (MCMC) methods. Hence, this study aimed to assess the TTR from severe acute malnutrition and its predictors among children aged 6–59 months via a Bayesian joint modeling framework.

## 2 Methods and Materials

### 2.1 Study Settings, Design and Period

An institution‐based retrospective follow‐up study was conducted from April 1, 2020, to May 30, 2024. The study was conducted at Felege Hiwot, Tibebe Ghion, Debre Tabor, Debre Markos, and Gondar, Comprehensive Specialized Hospitals (CSHs), which are located in Northwest Ethiopia, Amhara National Regional State. The data for this study were collected between July 1 and 30, 2024.

### 2.2 Population

All children with SAM aged 6–59 months who had received treatment at referral hospitals in Northwest Ethiopia composed the source population.

All the children with SAM were aged 6–59 months and had received treatment at the Felege Hiwot, Tibebe Ghion, Debre Tabor, Debre Markos, and Gondar CSHs from April 1, 2020, to April 1, 2023. However, incomplete records that lacked sociodemographic information on comorbidities; routine medications; patient treatment outcomes (i.e., cure, death, not recovered and defaulter); and those whose date of admission was not recorded were excluded.

### 2.3 Sample Size Determination and Sampling Procedures

Sample size determination for the survival outcome in this study was based on the following formula (Collett 2003):

$$n=\frac{4{\left(Z_{a/2}+Z_{1-\beta }\right)}^{2}}{P\theta_{R}^{2}}$$

where ***n*** is the required number of patients included in the study, α is the level of significance, set at 5%, 1−*β* is the power of the test, set at 80%, ***P*** is the probability of patients expected to recover, with a value of 0.519, and $\theta_{R}=\ln(\text{HR})$ is the log of the hazard ratio (HR) for the exposure variable (anemia), with a value of 0.44 on the basis of the study conducted in Bahir Dar City (Asres et al. 2018). The calculated sample size was 314, and after adding 5% incompleteness and a 1.5 design effect, the final sample size required for this study was 495.

**Figure 1:**
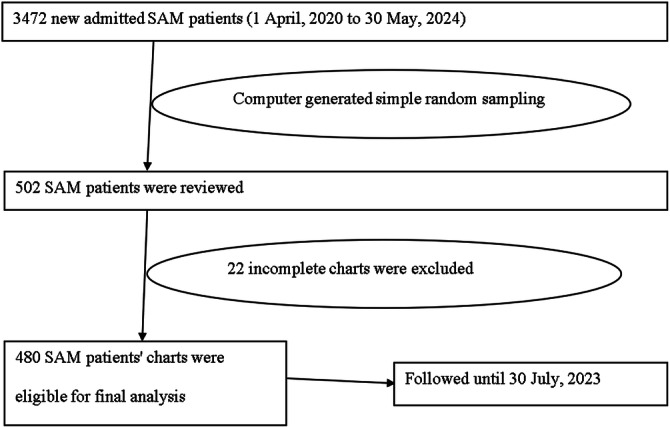
Sampling procedure flow chart of SAM patients aged 6–59 months on treatment at Amhara region referral hospitals, Ethiopia, 2020–2023.

The study was conducted in the Amhara region, which has eight referral hospitals. Five of these hospitals were randomly selected for participation. The sample size was proportionally distributed across the selected hospitals, and patient charts were chosen via a computer‐generated simple random sampling method. Among the five hospitals, there were a total of 3472 newly admitted SAM patients: University of Gondar Comprehensive Specialized Hospital: 926 SAM patients (132 selected), Felege Hiwot Referral Hospital: 747 SAM patients (107 selected), Debre Tabor Referral Hospital: 632 SAM patients (90 selected), Tibeb Gion Referral Referral Hospital: 573 SAM patients (82 selected), and Debre Markos Referral Hospital: 592 SAM patients (85 selected). A sampling frame was prepared by compiling the identification numbers of SAM patients from hospital registration records. The final selection of participant charts was performed via a computer‐generated simple random sampling method (Figure 1).

### 2.4 Variables of the Study

In this study, the dependent variable for the longitudinal submodel was weight, measured in grams. This variable was recorded from the start of treatment (baseline) and repeatedly measured every day, whereas the dependent variable for the survival submodel was the time from the date of SAM diagnosis to the time the child recovered/discharged, measured in days. The outcome was coded as censored (0) or event (1).

Independent variables: These include sociodemographic characteristics such as the age of the child, sex of the child, residence, comorbidities, types of comorbidities, and routine medications.

### 2.5 Operational Definitions

Survival time is the time in days from the child being diagnosed with SAM to the occurrence of the outcome (recovered/censored).

Event (recovered): Recovery of children from SAM or when the children fulfilled the discharge criteria determined by the ward physician.

The patient was discharged when the MUAC was ≥ 12.5 cm, and there was no bilateral pitting edema, and the patient was clinically well and alert.

Censored: These were those children who had not developed an event or those who had not recovered from SAM (death, defaulter, nonresponder, transferred‐out) at the end of the follow‐up period. Death is when the child dies while on treatment for SAM, and the documented death is confirmed by the physician. A defaulter is when the child is absent for two consecutive days. The child is transferred to another health facility for further medical care(Government of Ethiopia, Federal Ministry of Health 2019). A nonresponder is a patient who remains in treatment at the stabilization center (SC) and does not reach the SAM discharge criteria after 16 weeks (4 months) of treatment. Transferred out is defined as being moved to another facility for further medical care or being moved to receive care in another SC.

Kwashiorkor: Kwashiorkor is a severe form of acute malnutrition characterized clinically by bilateral pitting edema, a distended swollen abdomen, hair changes (thinning or reddish discoloration), and dermatosis.

Marasmus: This is a severe form of acute malnutrition characterized by an emaciated physical appearance/severe wasting and dry/loos skin appearance.

Marasmus‐kwashiorkor: This is a mixture of both kwashiorkor and marasmus (clinically characterized by bilateral pitting edema with sever wasting).

Comorbidities: Any disease condition (acute or chronic) present at admission in addition to SAM, which includes pneumonia, tuberculosis, diarrhea, anemia, vomiting, and retroviral infection.

### 2.6 Data Collection Procedures and Quality Assurance

A structured English‐language data collection checklist was developed on the basis of existing medical records of SAM patients and previous similar studies. To ensure accurate data extraction, ten nurses with experience in the SCs as data collectors and five health officers as supervisors were involved in the data collection process. To maintain data quality, the data extraction tool was pretested for consistency and completeness using 5% of patient charts 1 week before the actual data collection period. Necessary adjustments were made for the final data collection sheet by excluding variables not documented in the charts, such as educational status and smoking history. A 1‐day training session was conducted for the data collectors prior to data collection. The training covered the study objectives, data extraction procedures, and use of the data extraction form. Each component of the tool was explained clearly to the data collectors. Throughout the data collection period, the data extraction process was closely monitored by supervisors and the principal investigator. After each data extraction form was completed, the completeness of the data was checked, and corrections were made before the patient charts were returned.

### 2.7 Statistical Analysis

Data entry was performed via the Kobo toolbox, and the data were then exported to R software version 4.5.1 for further data cleaning, editing, and analysis. For continuous variables, the means with standard deviations (SDs) for normally distributed data and medians with interquartile ranges (IQRs) for nonnormally distributed data were used to describe the population. Categorical variables are described in terms of frequencies and percentages.

Survival time was calculated from the date of admission to either the date of events of interest (recovery from SAM) or censoring in days. Accordingly, the incidence density of recovery was computed as the number of new cases (recovered) divided by patient‐days at risk (total person‐days observation). The Kaplan–Meier (KM) method and the log‐rank test were used to compare recovery status between groups.

Cox PH and three parametric models (Weibull, exponential and log logistic) were fitted to identify risk factors. The best‐fitting models were selected on the basis of the Akaike information criterion (AIC). Accordingly, the Cox PH model was the preferred model for modeling TTR among SAM patients in the study area. To investigate the effect of the candidate covariates on the time‐to‐cure from SAM, we first performed univariate Cox regression analysis. Variables with *p* values less than 0.25 from the univariable analysis were included in the multivariable Cox regression analysis and further evaluated in the joint model. The Cox proportional hazard (PH) assumption was checked graphically (log–log plot) and statistically (Schoenfield residuals test) before the survival submodel was fit.

To account for the effect of an endogenous time‐varying covariate (weight) on the TTR, the true unobserved value of weight in the survival model was used. The trajectory of weight over time was approximately normally distributed. Before the linear mixed model (LMM) for the longitudinal submodel was fitted, exploratory analysis was conducted to visualize the patterns of individual profiles and average evolution changes graphically. A linear mixed effect model with a random intercept only and both a random intercept and slope was fitted. Consequently, a linear mixed effect model with both a random intercept and slope was chosen because it appropriately predicted the average change in weight measurements over time. To estimate the effects of longitudinal weight change on the TTR, the complete true history of weight for each subject was determined via a linear mixed‐effect model constructed by considering the effects of baseline covariates on weight evolution.

Finally, Cox PH with a linear mixed effect model was jointly modeled via the Bayesian approach. The joint models were subsequently fitted with different parameterizations via the JMbayes2 package of R software. The best parameterization for our data was selected via Deviance Information Criterion (DIC), Watanabe–Akaike Information Criterion (WAIC), and LP (Log Posterior Predictive Density or Log Probability) from different parameterizations of Bayesian joint models. The association parameter (alpha value) from the fitted joint model was used to assess the association between longitudinal biomarker (weight) change and TTR. In a multivariable Bayesian joint model, a 95% credible interval was used to determine a statistically significant factor. To assess the goodness of fit of the joint model, we used standardized marginal residuals for the longitudinal and martingale residuals for the survival submodel.

Our analysis treated death, default, non‐response, and transfers as a noninformative right censoring. The estimand of interest was the conditional risk of recovery among children who remain alive and under observation–conditional estimand. The validity of the assumption requires that censoring is unrelated to recovery prognosis. We therefore acknowledge this limitation and advise readers to interpret our results with caution.

### 2.8 Model Specification

#### 2.8.1 Longitudinal Submodel

Linear mixed‐effects (LME) models are generally used to model continuous longitudinal data (Verbeke et al. 1997). To measure the effect of the longitudinal covariates on the risk of an event, it is necessary to estimate the true unobserved values of the longitudinal covariates$\;\eta_{i}\left(t\right),$ and reconstruct the complete longitudinal history, $M_{i}\left(t\right),$ for each subject. This is achieved by postulating a suitable mixed‐effects model to describe the subject‐specific time evolution, as represented by the following notation (Diggle 2002).

$$\left\{\begin{array}{l} Y_{i}\left(t_{ij}\right)=\eta_{i}\left(t_{ij}\right)+\epsilon_{i}\left(t\right),\\ \eta_{i}\left(t_{ij}\right)=x_{i}^{⊤}\left(t_{ij}\right)\beta +z_{i}^{⊤}\left(t_{ij}\right)b_{i},\\ b_{i}∼N\left(0,D\right),\;\epsilon_{i}\left(t\right)∼N\left(0,\sigma_{\varepsilon }^{2}\right) \end{array}\right.$$

where $Y_{i}$ is the observed longitudinal response for the $i^{\mathrm{th}}$ subject, $X_{i}(t_{\mathrm{ij}})$ is the design matrix for the fixed effects β, $Z_{i}(t_{\mathrm{ij}})$ is the design matrix for the random effects$\;b_{i}$, and D is the variance–covariance matrix of the random effects. The error terms,$\;\varepsilon_{i}\left(t\right),$ are assumed to be mutually independent, independent of the random effects, $b_{i}$, and normally distributed with a mean of zero and variance of${\;\sigma }_{\varepsilon }^{2}$.

#### 2.8.2 Survival Submodel

The hazard function of the survival model is used to explain the probability that the event has occurred by time t. A common approach for modeling time–event data in a joint model is the Cox proportional hazards model (Rizopoulos 2012; Hickey et al. 2016). In particular, $T_{i}^{*}$ is the true event time for subject i, and ${T_{i}\;=\min(T_{i}^{*},C}_{i})$ is the observed event time, which is defined as the minimum value between the true event time $T_{i}^{*}$ and the censoring time $C_{i}$. Furthermore, $\;\delta_{i}=I\left(T_{i}^{*}\leq C_{i}\right)$ denotes the event indicator, which is equal to 1 if failure is observed $\left(T_{i}^{*}\leq C_{i}\right)$ and 0 otherwise. We assume that both censoring and measurement times are noninformative. The Cox proportional hazard model expresses that the hazard of an event at time t is given by (Rizopoulos 2012; Hickey et al. 2016):

$$\lambda_{i}\left(t\right)=\;\lambda_{0}\left(t\right)\text{exp}\left(W^{Τ}\Upsilon \right)\;$$

where $\lambda_{0}\;$ is the baseline hazard function and where W is the matrix of baseline covariates with the corresponding vector of regression coefficients γ.

#### 2.8.3 Joint Model Formulation

A joint model of longitudinal and time‐to‐event data can effectively evaluate the impact of the longitudinal covariate, measured with error, on the time to an event of interest. In this study, we assessed the ability of a longitudinal biomarker, weight, to predict the likelihood of recovery from SAM. The joint model is specified formally as follows (Rizopoulos 2012):

$$\left\{\begin{array}{l} \eta_{i}\left(t\right)=x_{i}^{⊤}\left(t\right)\beta +z_{i}^{⊤}\left(t\right)b_{i}\\ h_{i}\left(t|M_{i}\left(t\right),W_{i}\right)=h_{0}\left(t\right)\text{exp}\left[\gamma^{⊤}w_{i}+f_{l}\left(\alpha_{l},W_{i},b_{i},M_{i}\left(t\right)\right)\right] \end{array}\right.$$

where $M_{i}\left(t\right)=\{\eta_{i}\left(s\right)|0\leq s<t\}$ represents the history of the true unobserved longitudinal process up to time t and where $W_{i}\;$ contains baseline covariates with corresponding regression coefficientsγ. Similarly, the parameter α quantifies the effect of the underlying longitudinal outcome on the risk for an event.

While standard survival models such as the Cox proportional hazard model can identify baseline predictors of TTR (e.g., demographic or clinical factors at admission), they cannot directly incorporate the effect of evolving patient health status during treatment. In pediatric SAM recovery, a child's condition is not static; key clinical indicators, particularly weight, change dynamically throughout the therapeutic feeding program (TFP). The separate survival analyses treat weight as a baseline predictor, failing to capture how an individual child's weight trajectories influence their time to discharge.

### 2.9 Bayesian Estimation and Convergence Diagnostic

In this study, to estimate the unknown parameters, we adopt a fully Bayesian approach via the MCMC method, as it offers several advantages. This method provides full probability inference, allowing for the quantification of uncertainty through credible intervals (CrIs), which are often more intuitive than traditional confidence intervals. Furthermore, the Bayesian framework is particularly robust for complex models such as joint models, as they rely less on large sample asymptotic approximations and can more effectively handle the complex error structures inherent in longitudinal and survival data. This approach, therefore, allows us to move beyond identifying baseline risk factors to understand the dynamic relationship between a child's changing nutritional status and their path to recovery. Model fitting is implemented with the help of the JMBayes2 package of R software version 4.5.1. The joint posterior density finally takes the form (Rizopoulos 2012):

$$p\left(\theta ;b_{i}\;|\;Y_{i},T_{i}\right)\propto L\left(Y_{i},T_{i}\;|\;\theta \right)p\left(\theta \right)$$

where the term $L(Y_{i},T_{i}|\theta )$ is the joint likelihood of the longitudinal and event‐time outcome data and where p(θ) denotes the prior information of the unknown parameters θ in the joint model. The term θ represents all the parameters to be estimated from the model, while $T_{i}$ denotes the event time, $Y_{i}$ represents the longitudinal data and $b_{i}$ represents the random effects as defined in the submodels. We need to consider prior distributions for all unknown parameters in the parameter space θ. Owing to the absence of prior information on the parameters of interest, weakly informative priors are assigned. Therefore, weakly informative priors for all the elements of θ were chosen.

The prior distributions of all the elements of β, γ and α are assumed to be normal, with a mean of 0 and variance of 1000. We used the inverse gamma prior with shape = 0.01 and scale = 0.01 for the precision parameters of survival and the longitudinal submodel. Regarding the baseline risk function, we utilized a piecewise constant hazard approach, which is the default specification in the JMBays2 package (Rizopoulos 2012). This approach partitions the follow‐up time into intervals and assumes a constant hazard parameter on each interval, with independent gamma priors assumed for the parameters of the baseline risk function. This provides flexibility in capturing the shape of the hazard over time without imposing a restrictive parametric form. Finally, an inverse‐Wishart prior distribution was used for the variance–covariance matrices (**D**).

The posterior distributions of the parameters do not yield standard forms and hence are sampled by an MCMC algorithm to obtain suitable estimates. To ensure the chain convergence and accuracy of the parameters, we used trace plots, density plots, autocorrelation functions, Monte Carlo (MC) errors, effective sample sizes (ESSs), and Gelman–Rubin statistics, as suggested by (El Adlouni et al. 2006; Brooks et al. 2011), and confirmed them. Inferences were made on the basis of a simulation of 50,000 iterations with burn‐in of 5000, chain 3, and thinning of 5.

### 2.10 Ethics Statement

Ethical approval for this study was obtained from the Institutional Review Committee (IBR) of the University of Gondar, Gondar, Ethiopia, with reference number/IPH/2525/04/23/2023. The study was conducted in accordance with local legislation and institutional requirements. Written informed consent for participation was not required from the participants or the participants' legal guardian/nextguardian/next of kin in accordance with the national legislation and institutional requirements. No potentially identifiable images or data are presented in this study.

## 3 Results

### 3.1 Sociodemographic Characteristics of the Study Participants

A total of 480 children aged 6–59 months with SAM were included in this analysis. Among these, the majority, 344 (71.66%), were aged between 6 and 23 months. Above half, 251 (52.29%) were males, and 266 (55.4%) children were from rural areas. With respect to comorbidities, pneumonia in 196 (40.83%) children and diarrhea in 175 (36.46%) were the most common comorbidities, followed by vomiting in 113 (23.54%) children, whereas HIV/AIDS in 43 (8.96%) children was the least common comorbid condition, followed by tuberculosis in 51 (10.63%) children (Table 1).

**Table 1:** Baseline sociodemographic characteristics and comorbidities of children admitted to referral hospitals in the Amhara region, Ethiopia, 2020–2023.

Variables	Category	Survival status		Total *n* (%)
		Recovered (%)	Censored *n* (%)	
Age	6–11	76 (15.83)	90 (18.75)	166 (34.58)
	12–23	114 (23.75)	64 (13.33)	178 (37.08)
	24–35	53 (11.04)	18 (3.75)	71 (14.79)
	36–59	48 (10.00)	17 (3.54)	65 (13.54)
Sex	Female	131 (27.29)	98 (20.42)	229 (47.71)
	Male	160 (33.33)	91 (18.96)	251 (52.29)
Residence	Rural	161 (33.54)	105 (21.88)	266 (55.42)
	Urban	130 (27.08)	84 (17.50)	214 (44.58)
Comorbidities				
HIV/AIDS	Negative	268 (55.83)	169 (35.21)	437 (91.04)
	Positive	23 (4.79)	20 (4.17)	43 (8.96)
Pneumonia	No	197 (41.04)	87 (18.13)	284 (59.17)
	Yes	94 (19.58)	102 (21.25)	196 (40.83)
Tuberculosis	No	258 (53.75)	171 (35.63)	429 (89.38)
	Yes	33 (6.88)	18 (3.75)	51 (10.63)
Diarrhea	No	185 (38.54)	120 (25.00)	305 (63.54)
	Yes	106 (22.08)	69 (14.37)	175 (36.46)
Vomiting	No	249 (51.88)	118 (24.58)	367 (76.46)
	Yes	42 (8.75)	71 (14.79)	113 (23.54)
Anemia	No	254 (52.92)	106 (22.08)	360 (75.00)
	Yes	37 (7.71)	83 (17.29)	120 (25.00)

### 3.2 Clinical and Treatment Characteristics

Treatment of admitted patients with severe acute malnutrition in STCs was performed as per the national guidelines. This study revealed that 425 (88.54%) patients had been given routine medication during inpatient treatment. Most participants consumed vitamin A (82.1%), folic acid (76.0%), or therapeutic foods (RUTF: 69.0%; F‐75 formula milk: 93.3%; F‐100 formula milk: 72.9%) (Table 2).

**Table 2:** Clinical and treatment characteristics of 6–59‐month‐old children with SAM at referral hospitals in the Amhara region, Northwest Ethiopia, 2020–2023.

Variables	Category	Survival status		Total *n* (%)
		Recovered *n* (%)	Censored *n* (%)	
Vitamin A	No	51 (10.63)	35 (7.29)	86 (17.92)
	Yes	240 (50.00)	154 (32.08)	394 (82.08)
Folic Acid	No	73 (15.21)	42 (8.75)	115 (23.96)
	Yes	218 (45.42)	147 (30.63)	365 (76.04)
RUTF	No	89 (18.54)	60 (12.50)	149 (31.04)
	Yes	202 (42.08)	129 (26.88)	331 (68.96)
F‐75 formula milk	No	11 (2.29)	21 (4.38)	32 (6.67)
	Yes	280 (58.33)	168 (35.00)	448 (93.33)
F‐100 formula milk	No	94 (19.58)	36 (7.50)	130 (27.08)
	Yes	197 (41.04)	153 (31.87)	350 (72.92)
Deworming	No	200 (41.67)	127 (26.46)	327 (68.13)
	Yes	91 (18.96)	62 (12.92)	153 (31.87)
IV Fluids	No	226 (47.08)	149 (31.04)	375 (78.13)
	Yes	65 (13.54)	40 (8.33)	105 (21.88)
Blood transfusion	No	244 (50.83)	153 (31.87)	397 (82.71)
	Yes	47 (9.79)	36 (7.50)	83 (17.29)
IV antibiotics	No	35 (7.29)	20 (4.17)	55 (11.46)
	Yes	256 (53.33)	169 (35.21)	425 (88.54)

Abbreviations: IV = intravenous, RUTF = ready‐to‐use therapeutic food.

### 3.3 Exploring Weight Change

**Figure 2:**
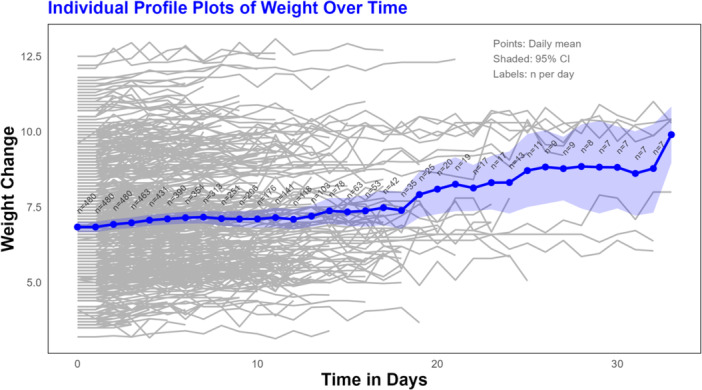
Individual profile plots for longitudinal outcomes (weight change) among SAM children aged 6–59 months under treatment in the Amhara region, CSRH, 2020–2023.

To understand the association between the weight measurement and time, individual profile plots were employed. To explore the mean change in weight measurement over time, we calculated day‐specific average weight with 95% confidence intervals (CIs) and sample size, instead of using LOESS smoothing, which can be biased by differential follow‐up. This approach reveals the true average weight trajectory and the effect of attritions over time. In Figure 2, the gray lines indicate individual child trajectories, blue line represents the day‐specific mean weight, the shaded ribbon indicates the 95% CIs, and the labels (“n = ”) show number of observations per day, respectively (Figure 2).

As shown in the plots, the individual profile plot revealed between‐variation changes in weight measurements over time. The weight trajectories of weight measurement for patients indicate significant variations in value at baseline. This suggests that a model with a random intercept is a good starting point.

**Figure 3:**
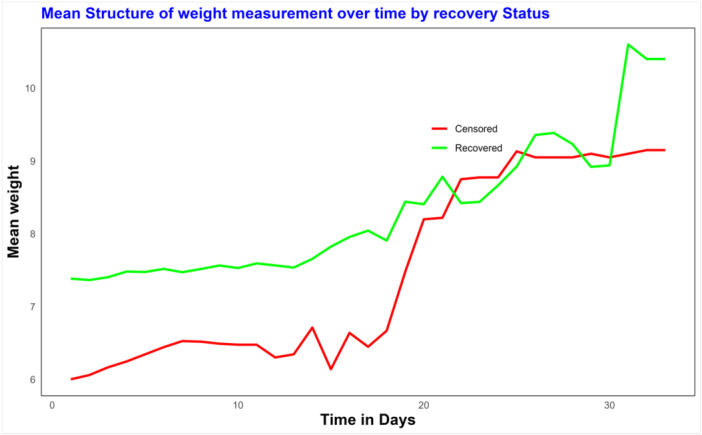
Mean profile plots of weight change by recovery status for SAM patients in selected referral hospitals, Northwest Ethiopia, 2020‐–2023.

The red and green curves, respectively, display the mean weight profiles for the recovered (event) and censored groups. The fact that the mean weight change of the event group was greater than that of the censored group up to some point suggests that there was a relationship between increased weight change and a greater likelihood of recovery (Figure 3).

### 3.4 Sensitivity Analysis for Missing Data

When weight measurements were not observed during a 24‐h period, these values were considered missing. In this study, 56 (11.7%) participants had at least one missing weight measurement and were thus excluded from the complete‐case analysis. A key challenge in handling missing data in longitudinal studies is that the observed data alone cannot distinguish between a missing at random (MAR) and a missing not at random (MNAR) dropout mechanism. To address this issue, we conducted a sensitivity analysis to examine the impact of different missing data handling methods on our results. Specifically, we compared parameter estimates and standard error estimates to assess the robustness of our findings. The results showed that the parameter estimates from the complete case analysis were not significantly different from those obtained via multiple imputations. Consequently, we chose to proceed with the complete case analysis approach within the Bayesian joint model framework, utilizing the current value parameterization, as outlined in Table 3.

**Table 3:** Comparison of the Bayesian joint model with the current value parameterization under multiple imputation and complete case analysis missing handling methods.

Methods	Parameterizations	WAIC	DIC
Complete case analysis	Current value	23,665.75	22,084.22
Multiple imputation	Current value	43,327.36	49,597.43,

*Note:* WAIC and DIC agree that the Bayesian joint model with the current value parameterization with complete case analysis missing handling approach has better predictive ability.

### 3.5 Incidence and Time to Recovery From Severe Acute Malnutrition

**Figure 4:**
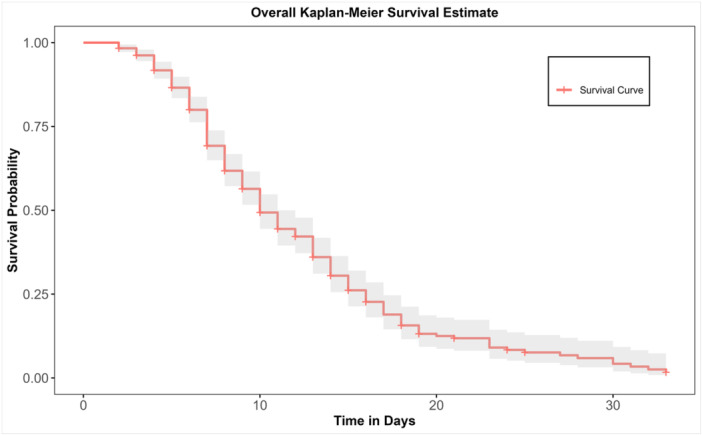
Plot of the overall estimate of Kaplan–Meier survival for time to recovery among SAM patients under treatment at Amhara region referral hospitals, 2020–2023. The blue solid line indicates the survival estimate, the dotted blue lines surrounding the survival estimate indicate the 95% confidence interval of the estimate, and the vertical red line indicates the median recovery time.

A total of 480 children aged 6–59 months were followed for 4356 person–days of observation (PDO). At the end of the follow‐up, 291 (60.6%) patients had recovered, with an incidence rate of 6.69 cases per 100 PDOs (95% CI: 5.93–7.47). The median recovery time from severe acute malnutrition was 10 days (IQR: 7–16) (Figure 4).

### 3.6 Treatment Outcomes

At the end of the follow–up period, 291 (60.63%) patients were classified as recovered. Defaulters accounted for 82 (17.08%), while 65 (13.54%) died and 42 (8.75%) transferred out (Supporting Information S1: Table S1).

### 3.7 Predictors of the Time to Recovery From Severe Acute Malnutrition

After the most suitable separate model was determined for the data, the proposed joint model was applied with the aim of investigating the effects of weight measurements on the TTR from the SAM under Bayesian inference. Joint modeling provides associated factors for both changes in weight and the TTR. The results showed that the current value of the weight trajectory was significantly associated with the hazard of recovery. Table 3 reveals that there is a strong prognostic association between longitudinal biomarkers (weight trajectory) and the time to recover (*α* = 0.136, 95% CrI: [0.042–0.232]), meaning that among children who remain under observation, a higher current weight value is associated with 1.15‐fold higher instantaneous rate of recovery at any time t(exp[0.136]=1.15,95%CrI:[1.04–1.26]). Importantly, this association is prognostic rather than causal: weight trajectory serves as a marker of clinical improvement and physiological status, not an independent mechanistic driver of recovery.

In addition, HIV status, anemia at admission, edema at admission, vomiting at admission, folic acid administration, F‐100 formula milk, and antibiotic use were significant predictors of the TTR. This study revealed that children who were HIV seropositive had a 54% lower hazard of recovery than did those who were HIV seronegative (AHR = 0.46, 95% CrI: [0.22, 0.90]). After adjusting for other variables in the model, the hazard of recovery was 52% lower for children with edematous malnutrition at admission than for those without edematous malnutrition (AHR = 0.48, 95% CrI: [0.32, 0.70]). Compared with their counterparts, children with anemia (AHR = 0.45, 95% CrI: [0.31, 0.64]) and vomiting (AHR = 0.62, 95% CrI: [0.44, 0.87]) were 55% and 38% less likely to recover from SAM, respectively. Surprisingly, after adjusting for other variables in the model, SAM children on folic acid supplementation had a 30% lower rate of recovery than their counterparts did (AHR = 0.70, 95% CrI: [0.53, 0.94]). Children who consumed F‐100 were 40% more likely to recover from severe acute malnutrition than were those without F‐100 formula milk (AHR = 1.4, 95% CrI: [1.08, 1.80]). Given that other variables in the model were constant, children who were on IV antibiotics had a 1.58‐fold faster recovery than those who were not on IV antibiotics did (AHR = 1.58, 95% CrI: [1.04, 2.46]), as presented in Table 4.

**Table 4:** Fitted Bayesian joint model result under current value parameterizations for SAM patients in the Amhara region of the CSRH, 2020–2023.

Variable	Category	Mean(γ)	MC error	AHR [95%CrI]	ESS	R‐hat
Child age	6–11					
	12–23	0.177	0.0025	1.19 [0.77, 1.84]	7060	1.00
	24–35	−0.418	0.0042	0.66 [0.39, 1.09]	3881	1.01
	36–59	0.029	0.0043	1.03 [0.62, 1.70]	3655	1.00
HIV/AIDS	Negative					
	Positive	−0.781	0.0038	0.46 [0.22, 0.90]*	9003	1.00
Pneumonia	No					
	Yes	0.047	0.0014	1.05 [0.79, 1.39]	10,715	1.00
Edema	No					
	Yes	−0.740	0.0017	0.48 [0.32, 0.70]*	14,091	1.00
Anemia	No					
	Yes	−0.793	0.0017	0.45 [0.31, 0.64]*	12,249	1.00
Vomiting	No					
	Yes	−0.477	0.0015	0.62 [0.44, 0.87]*	14,494	1.00
Folic acid	No					
	Yes	−0.351	0.0014	0.70 [0.53, 0.94]*	11,629	1.00
F‐75 formula milk	No					
	Yes	−0.533	0.0029	0.59 [0.32, 1.16]	1 3,470	1.00
F‐100 formula milk	No					
	Yes	0.334	0.0011	1.40 [1.08, 1.80]*	14,200	1.00
Intravenous fluid	No					
	Yes	0.260	0.0019	1.30 [0.90, 1.86]	9224	1.00
IV antibiotics	No					
	Yes	0.456	0.0021	1.58 [1.04, 2.46]*	10,650	1.00
Association						
Weight change	g/bod weight/day)	α=0.136	0.0007	1.15 [1.04, 1.26]*	4131	1.00

Abbreviations: β= Coefficient, CrI = credible interval, ESS = effective sample size, MC = Monte Carlo.

* = statistically significant.

## 4 Discussion

Despite various ongoing programs to improve children's nutritional status, severe acute malnutrition continues to be a global burden in many low‐ and middle‐income countries, especially in Sub‐Saharan Africa (Moyer et al. 2020).

This study assessed the predictors of TTR from severe acute malnutrition and its association with weight change among children aged 6–59 months in Ethiopia. This study revealed that the median TTR was 10 days (IQR: 7–16). The median TTR in this study was consistent with the median recovery time threshold set by the SPHERE standard in 2018 (Sphere Association 2018). The median TTR in this study was consistent with the median recovery time reported in studies conducted at Injibara University (Andargie and Zewdie 2024), Northeast Ethiopia (Feleke et al. 2024), the WagHimra Zone (Tadesse et al. 2021), Asosa (Bizuneh et al. 2022), Pawi (YIGEZU 2023), public hospitals in Northeast Ethiopia (Tefera et al. 2020), and Debre Markos and Fnote selam hospitals (Mekuria et al. 2017)., and Awi Zone (Injibara, Chagini, Dangila, and Gemjabet hospitals) (Workie et al. 2025). However, the median time of recovery in this study was far faster than the median recovery time reported in Dire Dawa (61 days) (Atnafe et al. 2019), Arba Minch (49 days) (Gebremedhin et al. 2020), and southern Ethiopia (17 days and 26 days) (Desyibelew et al. 2017; Fikrie et al. 2019). This difference may be attributed to variations in the sociodemographic characteristics of the respondents and disparities in healthcare services across different facilities. Furthermore, this short median recovery time is explained by the specific discharge criteria (MUAC ≥ 12.5 cm, no bilateral edema clinically well) and standard inpatient SAM management protocols in the study hospitals rather than a fixed minimum stay.

The present study revealed an overall incidence (recovery) rate of 6.69 cases per 100 patients per day (95% CI 5.93, 7.45). Addis Ababa (4.6) (Bitew et al. 2020), Asosa (5.28) (Bizuneh et al. 2022), Eastern Amhara Hospitals (6.9) (Tefera et al. 2020) and Pawi, Benishangul Gumuz (5.3) (Wondim et al. 2020) reported similar results. However, it is significantly less common than Dire Dawa research (17.23 per 100 PDOs) (Atnafe et al. 2019) and Awi zone public hospitals (Injibara, Chagini, Dangila, and Gemjabet hospitals) (11.2 per 100 PDOs) (Workie et al. 2025). This may be because the study conducted at Dire Dawa had a larger sample size. In addition, this could be a result of the difference in the study subjects' socioeconomic profiles and the dissimilarity in the preparedness of healthcare facilities.

The findings of this study also revealed that the proportion of children who recovered after treatment was 60.6% (95% CI: 56.19, 64.89) under the primary analysis which conservatively treats all censored children as non‐recoveries. The recovery rate reported in this study was lower than the minimum 75% threshold set by the SPHERE standard in 2018 (Sphere Association 2018). However, a bounding analysis accounting for the 189 censored children (65 deaths, 82 defaulters, and 42 transfers) demonstrated that the true recovery proportion lies between 60.6% and 86.5% that meets the SPHERE threshold. Compared to studies that have been conducted in the Gursum District Somali region (81.7%) (Budul et al. 2020) and southern Ethiopia (87%) (Teferi, Lera et al. 2010), Tigray (75.9%) (Kidane et al. 2023), Injibara (82.8%) (Andargie and Zewdie 2024), and Kenya (73.3%) (Mbaya 2015), our estimate is broadly comparable. The observed discrepancies could be due to diverse reasons, including variation in the timing and season in which the studies were conducted, the level of maturity of the OTP program in the study settings and dissimilarity in the underlying determinants of malnutrition across the localities, and importantly, differences in how censored children—particularly defaulters and transfers—are handled analytically. We recommend that future studies should routinely report censoring patterns and conduct sensitivity analyses to facilitate valid cross‐study comparisons.

In this study, the TTR was affected by several factors, including HIV status, anemia at admission, edema at admission, vomiting at admission, folic acid administration, F‐100 formula milk, and IV antibiotic use. Compared with children with marasmus, children with edematous malnutrition had a shorter recovery time. This finding is supported by other studies conducted in Ethiopia (Desyibelew et al. 2017; Baraki et al. 2020; Bizuneh et al. 2022; Aye et al. 2023) and India (Singh et al. 2014), implying that children with kwashiorkor and a mixed form of malnutrition are at greater risk of delayed recovery than are children with marasmus alone. This could be because these forms of malnutrition are primarily attributed to protein deficiency. Therefore, children with protein deficiency could have poor immunity, high fluid retention, poor appetite, and poor hormonal secretion. As a result, they feed poorly, develop metabolic problems, contract various infections, and may result in delayed recovery (Desyibelew et al. 2017; Bizuneh et al. 2022).

Children with comorbidities such as HIV/AIDS, anemia, and vomiting had slower recovery rates than did those without comorbidities. This was stated in a previous study, as children with comorbidities, in general, have a slower recovery time (Baraki et al. 2020). The decelerated recovery time among patients with HIV/AIDS, in particular, is supported by several other studies (Baraki et al. 2020; Bizuneh et al. 2022). First, infectious diseases increase nutritional requirements, resulting in poor survival as a result of poor intake. Second, these communicable diseases cause micronutrient deficiency following poor gastrointestinal absorption as a result of diarrhea and gastrointestinal erosion, and even microorganisms can compete with patients' cells by consuming nutrients. Studies have shown that these micronutrients are indispensable for increasing immunity and accelerating survival. Furthermore, these nutrients are required to synthesize protein and other macronutrients, which could accelerate the recovery time (Bachou et al. 2006; Banbeta et al. 2015). Considering these findings together, it is possible to infer that SAM children with medical complications, unfortunately, cannot recover earlier than those without these complications. Furthermore, HIV infection impairs immunity and eventually eliminates immune cells, especially if exposure to the virus is sustained (Korencak et al. 2019; Sainz et al. 2022). Pathologic malnutrition processes, such as malabsorption, increased metabolic requirements, and growth hormone malfunction, increase susceptibility to infection and can all be directly linked to immune dysfunction (Bourke et al. 2016).

This study revealed that children receiving IV antibiotics had a 1.58‐fold accelerated recovery time compared with those not receiving IV antibiotics, supporting their prioritized use per WHO guidelines. This finding is consistent with findings from Ethiopia (Yebyo et al. 2013; Yadeta et al. 2024) and clinical trials (Trehan et al. 2016) of antibiotics, which have the potential to reverse hidden infections such as pneumonia or bowel bacterial overgrowth, and constitute a crucial part of SAM treatment in India.

This retrospective follow‐up study revealed that children who consumed F‐100 formula milk had a 1.4‐fold faster recovery rate than those who did not. The present evidence aligns with the findings of earlier studies (Fikrie et al. 2019; Bizuneh et al. 2021). The high recovery could be attributed to the fact that F‐100 contains high‐energy products with high nutrient contents.

The present study revealed that children who received folic acid supplementation recovered 30% more slowly than those who did not. All children admitted with severe malnutrition are presumed to be infected with severe anemia, and folic acid is advised for their treatment. This medication is generally given to children who have further complications, such as severe anemia, which may be attributed to a longer recovery time (Government of Ethiopia, Federal Ministry of Health 2019; WHO 2023). Improving recovery may require lowering the risk of complications and other comorbidities.

In the present study, we observed that the current value of the longitudinal weight was strongly associated with TTR from SAM. For children who remain alive and under observation, and for patients who have the same covariates in the model at baseline and who have the same underlying weight measurements at time t, a one‐unit higher in the current value of weight is associated with a 1.15‐fold higher instantaneous hazard of recovery at any given time t. This means children with a higher weight trajectory at any time *t* tends to have a higher probability of recovery soon thereafter. Although no studies have assessed the strength of the association between longitudinal weight change and the rate of recovery among SAM children via a Bayesian joint modeling approach, some studies have shown that weight gain is strongly related to faster recovery time from SAM. The scientific explanation for this association is clear; for children with marasmus, a certain amount of daily weight gain is necessary to recover as quickly as possible, since weight gain is one of the criteria for discharge. The present study is in line with studies performed in the cities of Bahir Dar (Asres et al. 2018) and Addis Ababa (Abdu et al. 2024).

The public health importance of the findings of this study was to provide information for health professionals and parents, such as the routine daily monitoring of weight as a biomarker, which provides meaningful prognostic information that can help differentiate patients with regard to SAM and prevent its progression to long‐term effects of SAM, such as reduced human capital later in life in terms of education, self‐esteem and cognitive function. In addition, this study provides evidence of the factors associated with the TTR. Therefore, it helps minimize complications and longer hospital stays and maximizes efforts to prevent problems. The public health importance of this study is to prevent disability, economic loss, and loss of productivity associated with longer hospital stays by identifying the variables significantly associated with TTR from SAM.

One of the strengths of this study was that it estimated the TTR and identified its predictors via advanced modeling techniques.

The main limitation of this study was that its retrospective nature restricted the inclusion of all potential predictors of the rate of recovery, such as certain sociodemographic and behavioral factors, which may lead to an underestimation of effects and individual variations in the TTR from SAM. The findings for treatment‐related variables such as F‐100 formula, IV antibiotics, and folic acid supplementation, which may be confounded by indications, represent associations rather than causation. Furthermore, as this study was facility‐based, it did not include children with SAM at the community level. Moreover, we treated deaths, default, nonresponse, and transfers as non‐informative right censoring to estimate conditional recovery probabilities among children who remain under observation. This assumption may be violated, as death may act as a competing event for recovery and default and transfer may be associated with a poorer clinical trajectory. We therefore defined our estimand explicitly as a conditional measure of treatment efficacy rather than absolute real–world effectiveness. Accordingly, readers should interpret our primary estimates with caution, and further studies are encouraged to incorporate these analytical methods. However, a key strength of this study is that it is the first in Ethiopia to apply a Bayesian joint modeling approach to investigate TTR and associated factors among children aged 6–59 months with severe acute malnutrition.

### 4.1 Recommendations

In light of these findings, we recommend the following:

- Health professionals have strengthened the routine daily monitoring of weight as a biomarker, which provides meaningful prognostic information that can help differentiate patients with regard to SAM complications and prevent its progression to long‐term effects, such as reduced human capital later in life in terms of education, self‐esteem and cognitive function.
- Health professionals should pay greater attention to children with SAM who are HIV positive, who are malnourished, and who have anemia and vomiting at admission as comorbidities.
- Finally, further studies on this topic, including factors such as parental sociodemographic and socioeconomic characteristics and the perceptions of caregivers regarding the problem, with a prospective approach are recommended.

## 5 Conclusion

The recovery rate reported in this study was lower than the minimum 75% threshold set by the national and international SPHERE standards. The median recovery time of 10 days was optimal. The unobserved true current value of weight change was significantly associated with the TTR from severe acute malnutrition. Additionally, HIV status, anemia, edema, vomiting at admission, folic acid supplementation, F‐100 formula milk, and IV antibiotic use were significant predictors of the TTR from SAM.

## Author Contributions

**Dejen Kahsay Asgedom:** data curation, formal analysis, methodology, resources, software, supervision, validation, writing – original draft, writing – review and editing. **Maru Feleke:** conceptualization, data curation, formal analysis, investigation, methodology, project administration, resources, software, validation, visualization, writing – original draft. **Abraham Walelegn Zelalem:** conceptualization, data curation, formal analysis, investigation, methodology, project administration, resources, software, validation, visualization, writing – review. **Solomon Keflie Assefa:** data curation, formal analysis, investigation, methodology, project administration, resources, software, validation, visualization, writing – review and editing. **Bewuketu Terefe:** data curation, formal analysis, funding acquisition, investigation, writing – review and editing. **Nebiyu Mekonnen Derseh:** data curation, formal analysis, funding acquisition, investigation, writing – review and editing. **Ayenew Molla Lakew:** funding acquisition, investigation, methodology, project administration, resources, supervision, validation, visualization, writing – review and editing.

## Funding

No funding was received for this work.

## Conflicts of Interest

The authors declare no conflicts of interest.

## Supporting information


**Table S1:** Sensitivity analysis of recovery proportion under different assumptions about censored children (n = 480).
