# Supplementary material for: Time to Recovery From Severe Acute Malnutrition and Its Predictors Among Children Aged 6–59 Months in Ethiopia: A Bayesian Joint Modeling Approach
Source: Matern Child Nutr. 2026 Jul 28;22(3):e70220. doi: 10.1111/mcn.70220 (PMC13408432; doi:10.1111/mcn.70220)
Supplement: Supplementary file 1 — Table S1: Sensitivity analysis of recovery proportion under different assumptions about censored children (n = 480). [file MCN-22-e70220-s001.docx]

**Supplementary Table S1: Sensitivity analysis of recovery proportion under different assumptions about censored children (n = 480)**

| **Assumptions** | **Deaths (n = 65)** | **Defaulters**  **(n = 82)** | **Transfers**  **(n = 42)** | **Recovered** | **Recovery proportion** |
| --- | --- | --- | --- | --- | --- |
| Lower bound (all censored = non-recovered) | Non-recoveries | Non-recoveries | Non-recoveries | 291 | 60.6% |
| Upper bound (non-death censored = recovered) | Non-recoveries | Recoveries | Recoveries | 415 | 86.5% |
